# Responsiveness and minimal important differences of common disability measures in people with depression and anxiety disorders

**DOI:** 10.3389/fresc.2025.1556390

**Published:** 2025-04-30

**Authors:** Edimansyah Abdin, Vanessa Seet, Anitha Jeyagurunathan, Sing Chik Tan, Muhammad Iskandar Shah Mohmad Khalid, Yee Ming Mok, Swapna Kamal Verma, Mythily Subramaniam

**Affiliations:** ^1^ Research Division, Institute of Mental Health, Singapore, Singapore; ^2^ Department of Mood and Anxiety, Institute of Mental Health, Singapore, Singapore; ^3^ Department of Psychosis, Institute of Mental Health, Singapore, Singapore

**Keywords:** WHODAS, SOFAS, SDS, responsiveness, minimal important differences (MID), depression, anxiety

## Abstract

**Background:**

The Sheehan Disability Scale (SDS), World Health Organization Disability Assessment Schedule 2.0 12-item version (WHODAS 2.0), and Social and Occupational Functioning Assessment Scale (SOFAS) are commonly used disability measures in patients with depression and anxiety disorders. The current study aimed to compare the responsiveness of these three disability measures and establish their minimal important differences (MID) in the same population.

**Methods:**

A total of 308 patients (M = 36.1, SD = 12.7) who were recruited from outpatient clinics and completed all measures at the two assessment points were included in the current study. The MID was estimated using a triangulation approach while the internal and external responsiveness was evaluated using standardized response mean and receiver operating characteristic curves, respectively.

**Results:**

The best MID estimates for the WHODAS, SDS, and SOFAS were three, four, and six points, respectively. The internal responsiveness analysis showed that all three disability measures were well responsive in patients with improved or stable Patient Health Questionnaire-8 and Generalized Anxiety Disorder-7 scores at the 6-month follow-up. Meanwhile, the external responsiveness analysis demonstrated that all three disability measures showed adequate responsiveness to improvement, with AUC values of at least 0.7. However, when improvement criteria incorporated MID, only WHODAS was found to be adequately responsive.

**Conclusion:**

The results of this study will be a helpful guide for clinicians to track and detect meaningful improvements in patient functioning, ensuring continued high-quality clinical care and management.

## Introduction

Depression and anxiety are the most common psychiatric disorders and were ranked among the top 25 leading causes of years lived with disability worldwide by the Global Burden of Diseases, Injuries, and Risk Factors Study (1). A systematic review of data reporting the prevalence of major depressive disorder and anxiety disorders has suggested a substantial increase in the prevalence and burden of depression and anxiety across age groups as a result of the COVID-19 pandemic (2, 3). Numerous studies have shown that depression and anxiety are often associated with serious role impairment and disability (4). It has been suggested that anxiety and depressive symptoms are significantly associated with disability across the lifespan, with older adults reporting higher levels of disability than younger adults (5). The WHO defines disability as “a difficulty in functioning at the body, person, or societal levels, in one or more life domains, as experienced by an individual with a health condition in interaction with contextual factors” (6). Longitudinal studies suggest that the relationship between depression, anxiety, and disability is reciprocal where depression and anxiety are strong predictors of increased disability (7), while other studies reported that disability is a risk factor for depression (8).

In clinical settings and research studies, disability has been used as an important measurement outcome in providing care for individuals diagnosed with depression and anxiety. Different scales have been used to measure disability. The Sheehan Disability Scale (SDS), World Health Organization Disability Assessment Schedule 2.0 12-item version (WHODAS 2.0), and Social and Occupational Functioning Assessment Scale (SOFAS) are common disability measures that have been used to determine the severity of impairment or evaluate the effectiveness of intervention programs in patients with depression and anxiety disorders (9–13). The SDS is a well-validated scale designed to assess disability among people with mental disorders. It consists of three critical domains including work/school, social, and family life (14). The scale has been widely used to measure the impact of a variety of mental disorders on disability in clinical trials (9, 10, 14). The WHODAS 2.0 is used in both clinical and general population settings. The scale is free to use, short, reliable, and easy to administer (5–20 min) (6). Finally, the SOFAS (15) is an interviewer-rated scale that measures social and occupational functioning.

Singapore is an island city-state in Southeast Asia with a multiethnic Asian population of approximately 5.8 million people in 2024. The population comprises Chinese (74.3%), Malays (13.4%), Indians (9.1%), and other ethnic groups (3.2%). In Singapore, the WHODAS 2.0, SOFAS, and SDS have also been used earlier in clinical settings and research studies (16, 17). However, to our knowledge, there is limited evidence comparing the responsiveness of these three measures in the same population. Furthermore, there is a gap in our knowledge of what constitutes a minimal important difference (MID)—defined as the smallest change in scores perceived by a patient as beneficial or harmful—of these measures among those with depression and anxiety. Hence, the current study aimed to compare the responsiveness of these three measures and establish their minimal important differences in people with depression and anxiety disorders.

## Methods

A longitudinal study was carried out on a convenience sample at the Institute of Mental Health (IMH) and the Community Wellness Clinics (CWC) in Singapore. Briefly, the IMH is the only tertiary psychiatric hospital in Singapore. The CWC is a community clinic that offers comprehensive and integrated care for patients including those with mental illness in Singapore. Participants were patients recruited from outpatient clinics who were diagnosed with depression or anxiety disorders, Singapore citizens and Permanent Residents (PRs), those literate in English, and those aged 21 years and over. Written informed consent was obtained from all study participants.

## Questionnaires

The 12-item WHODAS 2.0 is a self-administered questionnaire that assesses disability during the preceding 30 days. It is designed to measure six domains: mobility, self-care, cognition, getting along, life activities, and participation. Each item is rated on a five-point Likert scale reflecting the level of difficulty, starting with “no difficulty” and increasing in an ordered fashion to “mild,” “moderate,” “severe,” or “extreme or cannot do.” A simple scoring can be generated by assigning each of the items a value—“no difficulty” (0), “mild” (1), “moderate” (2), “severe” (3), and “extreme or cannot do” (4). The scores are then summed up with a total ranging from 0 to 48.

The SDS is a self-rated generic scale that measures disability in three domains: work/school, social life, and home/family responsibilities (18). Each domain is measured with a single item using a Likert scale ranging from 0 (not at all) to 10 (extremely). The total scores can be generated by summing the three items (range, 0–30), with higher scores denoting greater functional impairments.

The SOFAS is an interviewer-rated scale used to measure social and occupational functioning in all patients. This scale provides a single-item rating of current functioning, with total scores ranging from 0 to 100. Higher scores denote better functioning (15).

The eight-item Patient Health Questionnaire (PHQ-8) is a self-administered depression scale used to measure symptoms in the past 2 weeks using a four-point scale, ranging from 0 (not at all) to 3 (nearly every day). The total scores range from 0 to 24, where scores of 10 and above indicate current depression (19).

The seven-item General Anxiety Disorder (GAD-7) scale is a self-administered scale used to measure anxiety symptom severity in the past 2 weeks. The items describe the most prominent diagnostic features of generalized anxiety disorder. GAD-7 scores range from 0 to 21 (20). Sociodemographic information including age, gender, ethnicity, and psychiatric diagnosis was also collected from the participants.

## Statistical analysis

Data are expressed as means and standard deviations for continuous variables and as percentages for categorical variables. In this study, we evaluated the MID and responsiveness of each measure following the COnsensus-based Standards for selection of health Measurement Instruments (COSMIN) recommendation (21). The PHQ-8 and GAD-7 were used as the reference measure, with changes in scale score between the baseline and 6-month follow-up determining subjects as improved, stable, or worse. Specifically, we defined the three groups as follows: “worse” if the change in PHQ-8 or GAD-7 scores over the 6-month follow-up was positive (6-month score − baseline score = positive), “stable” if the change in scores remained the same (6-month score − baseline score = 0), and “improved” if the change in scores was negative (6-month score − baseline score = negative). In this study, the PHQ-8 and GAD-7 were used as reference tools, as both are highly reliable and valid instruments commonly used in healthcare settings to track treatment response and measure the severity of depression and anxiety symptoms (22–24). Using a triangulation approach, the MID was estimated based on three metrics (25)—the standardized error of measurement (SEM), standard deviation (SD), and 6-month change scores by the PHQ-8 and GAD-7 scores. Responsiveness was first evaluated by correlating change scores across the WHODAS, SDS, SOFAS, PHQ-8, and GAD-7 using a Pearson's correlation analysis. Pearson's correlation coefficient was interpreted using the following criteria: >0.6, very strong; ≥0.5 to ≤0.6, strong; <0.5 to ≥0.3, moderate; and <0.3, weak (26). Subsequently, we estimated the internal and external responsiveness using standardized response means (SRMs) and receiver operating characteristic (ROC) curves, respectively. The SRMs were calculated by dividing the score change between the baseline and 6-month follow-up by the SD of the change score. The metric was compared against three groups: those who were improved, stable, and worse. The SRMs were interpreted using the following criteria: 0.2, 0.5, and 0.8 and above represent small, moderate, and large effects, respectively. We used ROC curves to assess the ability of the three questionnaires to correctly classify subjects as improved. Two types of improvements were analyzed: any improvement, defined as a positive change in the PHQ-8 and GAD-7 scores (6-month score − baseline score = positive), and improvement meeting the MID threshold criteria, defined as a positive change of five points and above in the PHQ-8 scores and four points and above in the GAD-7 scores. A four-point threshold for the GAD-7 was used based on a previous study by Toussaint et al. (27). Since no specific MID threshold exists for the PHQ-8, we have used a five-point threshold that has been estimated for the PHQ-9 (28, 29). An area under the curve (AUC) value of at least 0.7 was considered to indicate adequate responsiveness (30). All analyses were performed in Stata version 15.1 (StataCorp, USA) and RStudio software version 2022.07.2.

## Results

### Participant characteristics

The sociodemographic characteristics of the sample are summarized in Table 1. A total of 308 patients who completed all measures at both time points were included in the study. The mean age of the overall sample was 32.3 years (SD = 12.1). The sample comprised 46.4% male and 53.6% female respondents. The majority were Chinese (70.8), followed by Malays (14.6%), Indians (7.5%), and other ethnic groups (7.8%). Additionally, 54.9% (*n* = 169) of the respondents had depression, and 45.1% (*n* = 139) had anxiety. The mean (SD) PHQ-8 and GAD-7 total scores in this sample were 13.7 (5.2) and 12 (4.8), ranging from 5 to 24 and 5 to 21, respectively.

**Table 1:** Sociodemographic characteristics of the sample.

Variables	Mean	SD
Age	32.3	12.1
	*N*	%
Gender		
Male	143	46.4
Female	165	53.6
Ethnicity		
Chinese	216	70.8
Malay	45	14.6
Indian	23	7.5
Others	24	7.8
Diagnosis		
Depression	169	54.9
Anxiety disorders	139	45.1

### Correlation between change scores

The Pearson's correlation coefficient (*r*) for the changes scores between the baseline and 6-month follow-up among the WHODAS, SDS, SOFAS, PHQ-8, and GAD-7 scores are presented in Supplementary Table S1. The GAD-7 score correlated the most strongly with the PHQ-8 score (*r* = 0.68), followed by the correlation between the WHODAS and the SDS score (*r* = 0.55) and between the WHODAS and the PHQ-8 score (*r* = 0.52).

### MID

Table 2 summarizes the MID estimates for the WHODAS, SDS, and SOFAS. The reliability of the WHODAS and SDS was 0.9 and 0.8, respectively. By combining the three different metrics using the triangulating approach, we found that the best MID estimates for the WHODAS, SDS, and SOFAS were three, four, and six points, respectively.

**Table 2:** MID estimates of the WHODAS, SDS, and SOFAS.

Variables	WHODAS	SDS	SOFAS
No. of items	12.0	3.0	1.0
Observed range	0–40	0–30	25–85
Mean	15.2	14.7	57.8
SD	8.0	6.9	11.9
Reliability	0.9	0.8	N/A
Type of MID			
1 SEM	3.3	3.1	11.9
2 SEM	6.5	6.3	23.9
0.2 SD	1.8	1.4	2.4
0.35 SD	3.1	2.4	4.2
0.5 SD	4.5	3.5	6.0
Improved vs stable	2.2	4.1	5.9
Improved vs non-improved	6.8	6.7	7.8
Summary statistic of MID			
Range	1.8–6.8	1.4–6.7	2.39–23.88
Range, excluding high and low	2.64–5.51	2.76–5.11	5.04–9.86
Mean	4.0	3.9	8.9
Median	3.3	3.5	6.0
**Best estimate**	**3** **.** **00**	**4** **.** **00**	**6** **.** **00**

Bold values indicate the best estimate of MID.

### Responsiveness

**Figure 1:**
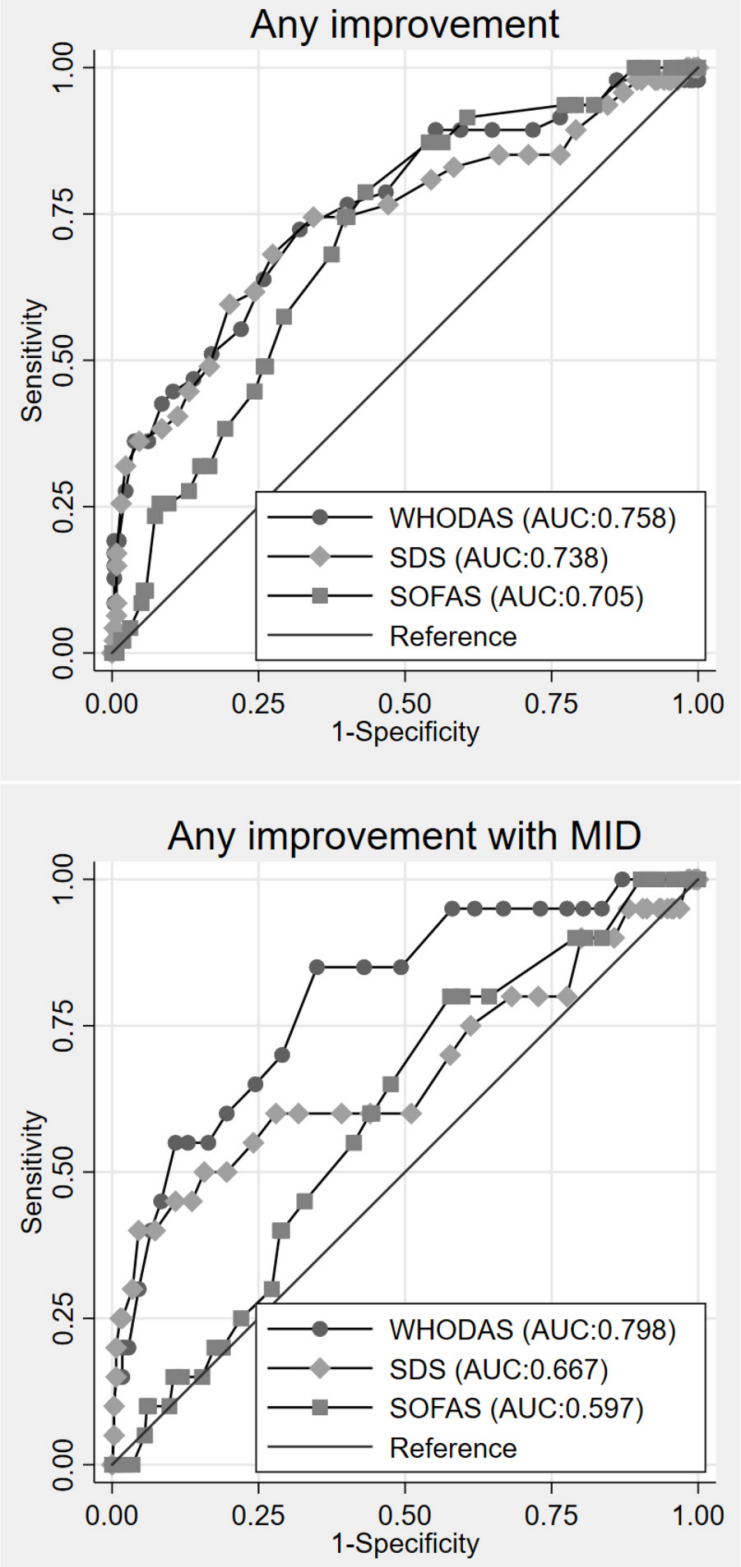
ROC curves for detecting any improvement and improvement with MID.

Table 3 summarizes the SRMs for the three measures. It shows that the SRMs were mostly of large magnitude (above 0.8) in patients who had improved or remained stable over the 6-month follow-up. For patients who had worsened over the 6-month follow-up, the SRMs were of small magnitude for the WHODAS and SDS and of moderate magnitude for the SOFAS. Table 4 shows the accuracy of the disability scales in detecting improvement. Meanwhile, Supplementary Table S2 presents the subgroup analysis of the accuracy of the disability scales in detecting improvement by diagnosis and gender. When we examined the responsiveness to improvement as a binary outcome (i.e., improved vs. non-improved), the changes in the WHODAS (AUC = 0.76) scores had the highest AUC value, followed by the SDS (AUC = 0.74) and SOFAS (AUC = 0.70). However, when MID thresholds of the PHQ-8 and GAD-7 were applied to the improvement criteria, only WHODAS was found to be adequately responsive (Figure 1, Table 4).

**Table 3:** Standardized response means (SRMs) of the WHODAS, SDS, and SOFAS.

Scales	Status	Baseline	6-month	Change	*p*-value	SRMs
WHODAS						
	Worse	14.86	15.25	−0.37	0.346	−0.06
	Stable	13.11	8.67	4.44	0.008	1.18
	Improved	17.70	11.11	6.60	0.000	0.82
SDS						
	Worse	14.45	12.82	1.63	0.000	0.23
	Stable	10.67	6.33	4.33	0.025	0.91
	Improved	16.57	8.19	8.38	0.000	1.01
SOFAS						
	Worse	58.27	64.22	5.95	0.000	−0.51
	Stable	61.22	69.11	7.89	0.027	−0.90
	Improved	54.36	68.15	13.79	0.000	−1.37

**Table 4:** Accuracy of the disability scales for detecting improvement.

	Accuracy for detecting any improvement						Accuracy for detecting improvement with MID					
Measure	AUC	95% CI		Sensitivity	Specificity	Optimal cutoff points	AUC	95% CI		Sensitivity	Specificity	Optimal cutoff points
All												
WHODAS	0.758	0.677	0.838	0.723	0.680	3	0.798	0.695	0.901	0.850	0.650	3
SDS	0.738	0.650	0.825	0.745	0.602	4	0.670	0.517	0.824	0.600	0.559	4
SOFAS	0.704	0.630	0.777	0.787	0.567	6	0.596	0.481	0.711	0.650	0.524	6

The area under the curve (AUC) is a probability of measure correctly differentiating between patients who have improved and those who have not.

## Discussion

This study presents several important findings. Based on a triangulation approach, the best MID estimates for the WHODAS, SDS, and SOFAS in this sample were three, four, and six points, respectively. The triangulation method has been widely used in prior studies to estimate minimal clinically important differences as this method involves the synthesis of clinical, statistical, and qualitative data to arrive at clinically relevant and statistically sound guidelines for interpretation (25, 31). Although a different approach was used, our findings on MID (three points) for the WHODAS in persons with depression and anxiety were in a similar range to those findings of the previous studies in patients with chronic musculoskeletal pain and chronic low back pain. For example, Wong et al. (32) estimated an MID of −3.22 for the WHODAS in persons with chronic low back pain. The MID was calculated using an anchor-based approach by considering the achievement of MID threshold improvement on the Short Form-36 Physical Functioning and Oswestry Disability Index (ODI). Meanwhile, Katajapuu et al. (33) estimated MID of 3.0 and 3.10 in patients with chronic musculoskeletal pain, respectively, when using distribution-based methods. In this approach, the MID was calculated using 0.33*SD and SEM criteria (33). Our findings were contrary to the findings of a previous study which found that the MID for the WHODAS was slightly higher (5.99 points) in adult patients with chronic low back pain (34).

To the best of our knowledge, this is the first study to perform a direct assessment of internal and external responsiveness of the WHODAS, SDS, and SOFAS in patients with depression and anxiety disorders. The current study evaluated internal responsiveness using SRMs, while external responsiveness was evaluated using AUC curves. Our internal responsiveness analysis showed that the three disability measures were well responsive in patients who had improved or stable PHQ-8 and GAD-7 scores at the 6-month follow-up. Meanwhile, among patients who became worse, only SOFAS was moderately responsive. In terms of external responsiveness analysis, the three disability measures responded well with adequate responsiveness—as determined by the AUC value of at least 0.7. Our AUC findings were in line with other studies. For example, the AUC value of the WHODAS in Kashin–Beck disease patients was acceptable (AUC = 0.71) (35), suggesting that the WHODAS is responsive to changes in our patients with depression and anxiety disorders.

This study had some limitations. First, the data for this study were collected from individuals diagnosed with anxiety disorders and depression in a single institution using convenience sampling. As the sample was predominantly of Chinese ethnicity, the findings may have limited generalizability to other settings and broader clinical population. It is possible that the responsiveness and MID of the WHODAS 2.0, SDS, and SOFAS scores may differ in other mental disorders. Another limitation of this study is that all measures were administered only in English. Hence, the responsiveness and MID among those who were not fluent in English remains uncertain as there could be significant differences both due to language issues and other social determinants. We used a combination of subjective self-reporting and objective interviewer ratings, which may have resulted in both recall bias and observer bias. While the WHODAS and SDS demonstrated good internal responsiveness to improvement, we also noted that the scales showed poor internal responsiveness in detecting deterioration. This limitation appears to be influenced by high skewness in both scales, which restricts their sensitivity to detect negative changes at the lower end of the spectrum. Hence, future studies with larger sample sizes and more balanced distribution across the full range of scales are needed to address this limitation. Additionally, the 95% confidence intervals were notably wide for the AUC. This variability warrants careful interpretation of the findings.

These limitations notwithstanding, to our knowledge, this is the first study that assesses responsiveness and MID in a single sample of patients with anxiety disorders and depression, enabling a direct comparison of responsiveness and MID metrics across these three disability measures. The results of this study will be a helpful guide for clinicians to track and detect meaningful improvement in patient functioning, ensuring continued high-quality clinical care and management.

## Data Availability

The datasets presented in this article are not readily available because raw data are unavailable for online access. However, readers who are interested in gaining access can contact the corresponding author. Access will be granted subject to the Institutional Review Board (IRB) and the research collaborative agreement guidelines. Requests to access the datasets should be directed to edimansyah_abdin@imh.com.s.
