# Supplementary material for: Responsiveness and minimal important differences of common disability measures in people with depression and anxiety disorders
Source: Front Rehabil Sci. 2025 Apr 30;6:1556390. doi: 10.3389/fresc.2025.1556390 (PMC12075521; doi:10.3389/fresc.2025.1556390)
Supplement: Supplementary file 1 [file Table1.docx]

Table 1 Sociodemographic characteristics of the sample.

| **Variables** | **Mean** | **SD** |
| --- | --- | --- |
| Age | 32.3 | 12.1 |
|  | **N** | **%** |
| Gender |  |  |
| Male | 143 | 46.4 |
| Female | 165 | 53.6 |
| Ethnicity |  |  |
| Chinese | 216 | 70.8 |
| Malay | 45 | 14.6 |
| Indian | 23 | 7.5 |
| Others | 24 | 7.8 |
| Diagnosis |  |  |
| Depression | 169 | 54.9 |
| Anxiety disorders | 139 | 45.1 |
